# Lesions to Primary Sensory and Posterior Parietal Cortices Impair Recovery from Hand Paresis after Stroke

**DOI:** 10.1371/journal.pone.0031275

**Published:** 2012-02-20

**Authors:** Eugenio Abela, John Missimer, Roland Wiest, Andrea Federspiel, Christian Hess, Matthias Sturzenegger, Bruno Weder

**Affiliations:** 1 Department of Neurology, Kantonsspital St. Gallen, St. Gallen, Switzerland; 2 Paul Scherrer Institute, Biomolecular Research, Villigen, Switzerland; 3 Support Centre for Advanced Neuroimaging (SCAN), Institute for Diagnostic and Interventional Neuroradiology, University Hospital Inselspital and University of Bern, Bern, Switzerland; 4 Department of Psychiatric Neurophysiology, University Hospital of Psychiatry and University of Bern, Bern, Switzerland; 5 Department of Neurology, University Hospital Inselspital and University of Bern, Bern, Switzerland; Cuban Neuroscience Center, Cuba

## Abstract

**Background:**

Neuroanatomical determinants of motor skill recovery after stroke are still poorly understood. Although lesion load onto the corticospinal tract is known to affect recovery, less is known about the effect of lesions to cortical sensorimotor areas. Here, we test the hypothesis that lesions of somatosensory cortices interfere with the capacity to recover motor skills after stroke.

**Methods:**

Standardized tests of motor skill and somatosensory functions were acquired longitudinally over nine months in 29 patients with stroke to the pre- and postcentral gyrus, including adjacent areas of the frontal, parietal and insular cortices. We derived the recovery trajectories of each patient for five motor subtest using least-squares curve fitting and objective model selection procedures for linear and exponential models. Patients were classified into subgroups based on their motor recovery models. Lesions were mapped onto diffusion weighted imaging scans and normalized into stereotaxic space using cost-function masking. To identify critical neuranatomical regions, voxel-wise subtractions were calculated between subgroup lesion maps. A probabilistic cytoarchitectonic atlas was used to quantify of lesion extent and location.

**Results:**

Twenty-three patients with moderate to severe initial deficits showed exponential recovery trajectories for motor subtests that relied on precise distal movements. Those that retained a chronic motor deficit had lesions that extended to the center of the somatosensory cortex (area 2) and the intraparietal sulcus (areas hIP1, hIP2). Impaired recovery outcome correlated with lesion extent on this areas and somatosensory performance. The rate of recovery, however, depended on the lesion load onto the primary motor cortex (areas 4a, 4p).

**Conclusions:**

Our findings support a critical role of uni-and multimodal somatosensory cortices in motor skill recovery. Whereas lesions to these areas influence recovery outcome, lesions to the primary motor cortex affect recovery dynamics. This points to a possible dissociation of neural substrates for different aspects of post-stroke recovery.

## Introduction

Stroke is one of the leading causes of long-term disability in adult life. Outcomes are highly heterogeneous, with 50–88% of survivors suffering from permanent sensorimotor sequelae, while others regain almost complete functionality. Many clinical predictors have been defined, but less is known about the effect of biological determinants, e.g. degree and localization of structural lesions, on longitudinal post–stroke recovery. Recent neuroanatomical studies in stroke patients show that the amount of corticospinal tract (CST) injury correlates well with chronic motor status [1], [2], but prospective studies that analyze lesion topography, its anatomical relationship to cortical structures and behavioural implications are missing. Over the past decade, a computerized probabilistic histological atlas of the human brain has been generated based on an observer-independent microscopic analysis of ten healthy post-mortem brains [3], [4]. This atlas contains templates that describe the probability of finding a specific cyto- or myeloarchitecture at a given location in a standard stereotaxic space. Two recent cross-sectional studies and one longitudinal study have used cytoarchitectonic information to infer structure-function relationships in patients with focal ischemic lesions [5], [6], [7].

Here, we applied probabilistic cytoarchitectonic maps to investigate the neuroanatomical substrates that determine the recovery of skilled hand function, a common deficit after stroke. Specifically, we asked whether cytoarchitectonically defined lesion patterns found in the acute stage are associated with different recovery trajectories in the long term. To answer this question, we investigated at regular intervals over nine months patients with hand paresis and lesions to the sensorimotor cortices, using a standardized measure of motor skill impairment. Patients with cortical stroke show a higher probability of regaining some individual movements than patients with subcortical stroke and thus seem better suited to observe recovery processes [8]. For the purpose of our study, we define two aspects of recovery: recovery outcome (whether a motor deficit persists or not) and recovery dynamics (how rapidly behavioral change occurs). Our primary aim was to analyse the impact of high lesion load of the somatosensory cortices on motor skill recovery, a topic rather neglected in the literature [9] and to test the hypothesis that sensory dysfunction would affect the capacity to re-establish dextrous hand function and thus the recovery outcome.

## Materials and Methods

### Subjects

We prospectively recruited patients at two comprehensive stroke centers (Departments of Neurology, University Hospital Bern and Kantonsspital St. Gallen, Switzerland) from January 01, 2008 through July 31, 2010. Inclusion criteria were: (1) first ever stroke, (2) clinically significant contralesional hand plegia or paresis as a main symptom, and (3) involvement of the pre- and/or postcentral gyri confirmed on diffusion-weighted (DWI) and fluid attenuated inversion recovery (FLAIR) MRI scans at that time. Additional involvement of frontal, parietal and opercular regions was accepted but not selected for. Patients were excluded if they presented (1) aphasia or cognitive deficits severe enough to preclude understanding the study purposes or task instructions, (2) prior cerebrovascular events, (3) occlusion of the carotid arteries in MR–angiography, (4) purely subcortical stroke, and (5) other medical conditions interfering with task performance. We recruited 36 patients, of which 7 dropped out (3 withdrew consent, 2 were too frail for repeated testing, 1 was shown to have no cortical stroke after enrollment, 1 was lost to follow-up). The final sample consisted of 29 patients (5 female). As a control group, we recruited 22 healthy older adults (11 female) from the local community. Groups were matched for age (unpaired two-tailed t-test: t (49) = 3.4, p<.12) and handedness according to the Edinburgh Handedness Questionnaire (unpaired two-tailed t-test: t (49) = 0.36, p<.30). The study received ethical approval from both research centers (Ethikkommission des Kantons St. Gallen (EKSG), Kantonsspital St. Gallen, 9007 St. Gallen and Kantonale Ethikkommission Bern (KEK), 3010 Bern, Switzerland) and all participants gave written informed consent before enrollment according to the Declaration of Helsinki.

### Behavioral Data

Baseline measurements were recorded in the first week after stroke (mean ± SD: 5.6±3.6 d post-stroke), followed by 9 monthly visits (30.0±9.6 d between examinations; 275.5±13.0 d of total follow-up). Healthy volunteers were tested twice a month apart (29.5±1.3 days between examinations). Tests were pseudorandomized across modalities, subjects, hands and visits (see Appendix S1 for details on testing procedures).

#### Clinical Assessment

Clinical severity of stroke was assessed at beginning of the study using the National Institutes of Health Stroke Scale (NIHSS) [10], [11].

#### Motor Assessment

Grip force (GF) was calculated as the average of three power grips using a Jamar hydraulic hand dynamometer [12]. Motor skill was measured at each hand separately using the Jebsen-Taylor Test (JTT), a standardized quantitative assessment that consists of seven timed subtests that simulate everyday activities [13]. In the present study, we included only data from those five JTT subtests that have shown the highest stability and test-retest reliability according to previous reports [13], [14]. These subtests were: (1) turning five index cards (“Turn”), (2) picking six small common objects (paper clips, bottle caps, coins) and dropping them into an empty can (“Pick”), (3) stacking four checkers on a board (“Stack”), (4) lifting and moving empty cans (“Light”), and (5) lifting and moving heavy cans (“Heavy”).

#### Somatosensory Assessment

Pressure perception thresholds (PPT) were measured with Semmes-Weinstein monofilaments (Senselab Aesthesiometer, Somedic AB, Hörby, Sweden) using a simple staircase algorithm to reduce testing time and subject fatigue [15]. Two-point discrimination (2PD) was measured using a graded caliper (2-point Discriminator, Medwork Instruments, Vancouver, Canada). Tactile object recognition (TOR) was tested using a standardized protocol with 30 everyday objects as previously described [16]. Impaired TOR was empirically defined as 10 or less correctly identified objects.

#### Recovery Modeling

Since we were interested in the recovery of skilled motor function, we focused our analysis on the time courses of the JTT subtests. Our procedures rested on the following considerations. First, we decided to analyze each subtest separately (instead of calculating the sum score usually employed), because during each of them the patient performs particular combinations of reaching, grasping and manipulating movements that require very different contributions from proximal and distal segments of the upper limb. Therefore, we hypothesized that the demands on motor control and the effects of injury and recovery would vary considerably between subtests. In our view, an additive score would thus represent a mixture of behavioral effects rather than reflect the recovery of a specific motor function. Second, we used an approach termed “response feature analysis” to characterize individual recovery. The idea behind this approach is to reduce the repeated observations on each patient to a statistic that captures essential features of his/her behavioral response over time [17], [18]. This can be achieved by fitting linear and non-linear curve models to each patient's longitudinal data and using the derived parameter estimates to represent individual response or recovery characteristics. Response feature analysis thus effectively reduces the problem of serially correlated measures and provides a simple way to investigate within-subject recovery characteristics [17], [18].

Importantly, a measure that represents recovery should show a strong longitudinal effect in the examined cohort. To quantify this effect, we determined for each JTT subtest whether it showed at the initial observation a deviation greater than 3 SD from the mean score calculated from the five last observations of a given patient. This procedure relied on the observation that behavioral scores are known to reach a plateau roughly 5 months post-stroke [19], [20], [21]. Additionally, we calculated for each patient the longitudinal within-subject variance of each subtest. Subtests that showed both a high frequency of >3 SD deviations and a large within-subject variance were classified as suitable recovery measures. In order to control for age, gender and hand dominance, patients' raw scores of each subtest were converted to z-scores using the mean and standard deviation of the corresponding scores from the healthy control group and plotted against time to visualize individual recovery trajectories. We then fit three models to each recovery trajectory: (1) a linear function *y = I+βt*, describing recovery at a constant rate (model Lin), (2) an exponential model y = I *exp ^(−βt)^ describing recovery with a time-dependent rate that converges to zero (model Exp), and (3) an exponential model y = I *exp ^(−βt)^+c describing recovery with a time-dependent rate that converges to a constant other than zero (model “ExpC”). In all models, *I* denotes the intercept (i.e. initial motor deficit), *β* the recovery rate, *t* time and *c* a constant that specifies the chronic motor deficit. The model parameter *β* thus describes recovery dynamics, and *c* recovery outcome. To determine which model best fit the data of each patient for a given task, we used a model selection procedure based on Akaike's information criterion with finite sample correction (AICc), a statistic that reflects the trade-off between likelihood and complexity of a model [22], [23]. AICc values can be transformed into conditional probabilities (or “Akaike weights”, *w*) that reflect the evidence for a model given the data and the set of evaluated models. The model with the lowest AICc value thus the highest yields the best fit to the data (Appendix S1). From previous research, recovery trajectories of motor functions are known to follow non-linear patterns [19], [20], [21]. Therefore, we predicted that models Exp and ExpC would be the most likely models for most patients. For each JTT task, patients were assigned to subgroups according to the model that best fit their recovery trajectory. Accordingly we describe the subgroups as follows: subgroup Lin, patients with fast complete recovery; subgroup Exp, patients with slow complete recovery; ExpC patients with impaired recovery. Note that depending on the task, a given patient might belong to different subgroups.

#### Statistical Analysis

All variables were tested for normality using the Shapiro-Wilk test. Nonparametric tests were applied where appropriate. The standard threshold of significance was chosen as p<.05 (Bonferroni corrected). For recovery trajectories, the range of normal motor performance was defined as z = 0±2.5. The significance threshold therefore was set to be z<−2.5 corresponding to p<.005 one-tailed (since patients were not expected to perform significantly better than healthy controls). Motor test scores below z = −2.5 thus indicate significant behavioral impairment, scores above this threshold normal motor performance.

### Imaging Data

#### Imaging Acquisition and Lesion Reconstruction

 Images were acquired during the first days after stroke (mean ± SD: 2.0±2.4 d; range 0–3 days, except patients no. 1: 6 d and no. 18: 13 d). Scanning was carried out using a 1.5 Tesla Siemens Sonata scanner for the first 9 patients, and 3 Tesla Siemens Trim Trio scanners for all others. For all patients, T1-weighted and DWI scans were acquired with standard sequence parameters (Appendix S1). We used the DWI scans for lesion definition because they show superior contrast for ischemic lesions compared to T1-images. T1-images were in turn used to calculate the necessary normalization parameters for transformation of all images into stereotaxic space, since they show superior anatomical detail (see below). We proceeded as follows: DWI and T1 scans were first coregistered using Statistical Parametric Mapping 8 (SPM8, http://www.fil.ion.ucl.ac.uk/spm/software/spm8/). Lesions were then manually traced in native space onto the DWI scans using MRIcron (http://www.cabiatl.com/mricro/mricron/index.html), yielding binary lesion maps. To avoid bias, lesion reconstruction was performed without reference to the results of the behavioral data analysis. Lesion maps and T1-images were then simultaneously spatially normalized to Montreal Neurological Institute (MNI) stereotaxic space using the unified segmentation algorithm in SPM8. Cost function masking, i. e. exclusion of lesioned voxels from the spatial normalization algorithm, was applied to prevent image distortions. To compensate for residual intersubject variability due to different field strengths, individual anatomical variability and coregistration errors, normalized lesion maps were smoothed with a 4 mm FWHM Gaussian kernel, masked at a threshold of >0.2 and resampled to 1.0×1.0×1.0 mm^3^ resolution (identical to the probabilistic anatomical atlas).

To estimate the accuracy of the normalization procedure, we measured the voxelwise standard deviation between the SPM8 single-subject T1 template and the normalized patient T1 scans. If the presence of lesions affects the normalization of injured brains, there should be areas of systematic increase in the voxelwise standard deviation map, especially in regions surrounding the central sulcus. Otherwise, the map would be expected to be homogenous. Furthermore, we calculated the average Euclidean distance between the non-lesioned “motor hand area” of each patient (visually identified as a characteristic “knob” of the precentral gyrus exhibiting the shape of an inverted omega) and the motor hand area of the single-subject T1 template (Appendix S2).

#### Lesion Subtraction Mapping

 Patients were grouped according to their modeled recovery trajectories. Lesion overlap maps for each subgroup where then generated using MRIcron. A core map for each subgroup was defined thresholding for voxels with a lesion frequency of at least 90%, thus representing the maximum overlap of lesions in this subgroup. The center of gravity of each core map was determined automatically in MRIcron. Additionally, a map of the complete cohort was generated including only voxels lesioned in at least 5 patients. The subgroup with impaired recovery (i.e. those patients whose recovery was best described by model 3) was chosen as the group of interest to which other subgroups were compared. Voxelwise subtractions between subgroups where performed by subtracting the percentage of patients without lesion in a voxel from the percentage with lesion in the same voxel. This yielded subgroup maps with values that ranged from +100% to −100%, where high values indicate voxels more frequently lesioned in the impaired subgroup. For lesion subtractions, only voxels with lesion frequency ≥50% were accepted as significant and subgroup maps were thresholded accordingly.

#### Cytoarchitectonic Mapping

 Lesions were mapped onto a probabilistic atlas using the Anatomy Toolbox for SPM8 (http://www.fz-juelich.de/inm/inm-1/spm_anatomy_toolbox) to identify the most likely cyto- and myeloarchitectonic areas involved [6]. The atlas is based on observer-independent histological analysis of ten post-mortem brains and contains maximum probability maps (MPMs) of major cortical and subcortical structures in MNI space [3]. MPMs are non-overlapping maps of voxels having the highest probability of belonging to a given area, and provide a reliable basis for cytoarchitectonic classification [4], [24]. We used the atlas to derive two measures: (1) Lesion extent, i.e. the intersection volumes between the binary lesion mask of each patient and the MPM of each cytoarchitectonic area affected by this lesion, and (2) lesion centrality, i.e. the topographical distribution of lesioned voxels on the complete map of each affected cytoarchitectonic area [25].The latter was calculated as the ratio of the mean probability for a given area within the lesion mask divided by the mean probability for the same area in the whole brain volume (“centrality ratio”, cr). The rationale for doing so is as follows: the center of a probabilistic cytoarchitectonic map is defined by voxels that belong to a given cytoarchitecture, e.g. area 4a, with high probability. Lesions towards the center of an area will include these voxels more frequently than could be expected from the overall probability distribution of the respective area. Put differently, voxels that have a high probability to belong to a given area will be overrepresented within a central lesion compared to the mean probability of the complete cytoarchitectonic map referred to the whole brain volume. Since the mean probability of an area across the whole brain is less than 1, cr will be greater than 1 for central lesions and less than 1 for peripheral lesions [25].

## Results

### Behavioral Data

Clinical characteristics, MRI lesion location and initial behavioral scores are summarized in Table 1 (for continuity, the order of patients is the same as in following tables). As additional clinical symptoms, 7 patients presented mild motor aphasia and 5 tactile neglect at baseline. These deficits had resolved by the fourth visit (3 months after stroke) in all cases. At baseline, 3 patients were plegic and could not perform the JTT; one could not perform the GF task. After excluding these scores, there were no differences at baseline between right and left hemispheric stroke patients in any of the motor and somatosensory assessments (two-sided independent samples Mann-Whitney test for GF: U = 86.5, p<.760, total score of JTT: U = 94, p<.574, PPT: U = 102.5, p<.892, 2PD: U = 81, p<.430 and TOR U = 90.0, p<.681). Also, neither clinical severity as assessed with the NIHSS (U = 105.5, p<.765) nor lesion volumes (U = 128.0, p = .250) were significantly different between right and left lesion groups. In healthy control subjects, JTT subtests were highly reproducible (Pearson's correlation coefficient *r* for Turn = 0.96, Pick = 0.98, Stack = 0.95, Light = 0.93, Heavy = 0.95, all p<.001), confirming previous reports [13], [14].

**Table 1:** Baseline demographic, neuroimaging and sensorimotor data.

No.	Age	Sex	LQ	Hem	NIHSS	Lesion Location°					GF	JTT	PPT	2PD	TOR
	(y)					PM	MI	SI	SII	PPC	(kg)	(s)	log[g/mm^2^]	(mm)	(n)
4	73	M	82	L	6		+	+		+	9	115.0	1.2	25	1
6	53	F	83	R	9	+	+	+		+	11	64.7	1.4	7	0
13	56	M	100	R	14	+	+	+	+	+	17	143.8	2.1	35	0
14	54	M	95	L	6	+	+	+	+		10	135.6	2.1	40	0
17	51	M	88	R	4		+	+	+		9	240.6	0.7	5	4
18	70	M	95	L	6			+	+	+	24	52.5	1.8	40	0
1	77	M	54	L	4		+	+			31	38.0	0.6	4	30
2	50	M	81	R	7	+	+				6	-	0.4	2	21
3	78	M	100	R	5	+	+	+			15	91.3	0.5	2	28
5	80	M	67	L	2	+	+				42	65.2	0.7	6	29
7	78	F	100	R	4		+	+	+		18	37.3	1.4	15	0
8	80	M	100	R	5		+	+	+		45	42.6	2.1	20	10
10	65	F	82	L	3			+	+		20	24.2	0.8	5	30
12	54	M	38	R	5		+				14	38.4	0.8	3	28
19	74	M	65	R	4		+	+	+	+	34	57.1	0.7	7	27
20	49	M	67	R	3		+	+		+	49	59.5	2.0	13	0
21	44	M	100	L	3		+	+			9	45.9	0.3	4	28
22	80	M	33	R	4		+	+			23	60.7	1.5	5	23
23	78	M	100	R	2		+				23	43.4	0.6	5	30
9	70	F	64	R	3			+	+	+	12	35.8	0.9	7	30
11	41	F	89	R	3		+	+	+		32	23.5	0.5	4	30
15	73	M	82	R	3		+				51	23.1	0.8	7	29
16	58	M	80	L	4			+	+		20	39.4	0.8	6	28
24	63	M	43	L	5		+	+		+	52	29.0	1.3	11	20
25	63	M	100	L	3		+				30	19.7	0.5	5	30
26	75	M	100	R	5		+	+			3	-	0.5	6	30
27	78	M	50	L	4		+				23	45.1	0.8	10	29
28	60	M	100	R	3		+				31	70.2	0.5	4	30
29	75	M	30	R	5		+	+			-	-	1.9	18	24
**Pat** *	**66.3 (11.6)**	**5 F, 24 M**	**82(65–100)**	**18 R, 11 L**	**4 (3–5)**	**6**	**24**	**21**	**10**	**8**	**22.1(14.3)**	**59.7 (48.3)**	**1.0 (0.6)**	**11.1 (10.9)**	**28 (1–29)**
**Ctr** *	**67.6 (6.5)**	**11 F, 11 M**	**88 (75–93)**								**35.7 (10.8)**	**23.5 (3.2)**	**0.3 (0.1)**	**4.2 (2.1)**	**30 (28–30)**

Note that patients are not ordered chronologically (No.), but according to the following tables 3 and 4 for continuity. The two lowest rows show descriptive statistics for patient (Pat) and control (Ctr) groups. Abbreviations: GF = grip force, Hem = affected hemisphere; LQ = laterality quotient for handedness according to the Edinburgh Handedness Questionnaire (where +100 denotes strongly right-handed); JTT = Jebsen Taylor Test score; MI = primary motor cortex (precentral gyrus); NIHSS = National Institutes of Health Stroke Scale; TOR = tactile object recognition; 2PD = two-point discrimination; PM = premotor cortex (superior and middle frontal gyri anterior to precentral gyrus); PPT = pressure perception threshold; PPC = posterior parietal cortex (superior and inferior parietal lobe); SI = primary somatosensory cortex (postcentral gyrus).

*Values are mean (SD) for ratio data, median (interquantile range) for ordinal data, and number for count data. ° As identified on routine diffusion-weighted and fluid-attenuated inversion recovery scans. -, data not available (task could not be performed).

### Recovery Measure and Trajectories

In 23 of 29 patients, initial scores of the subtest “Picking Small Objects” (“Pick”) were greater than 3 SD above the final plateau (Stack: 19/29, Heavy: 18/29, Turn: 16/29, Light: 16/29). Thus, “Pick” had the highest frequency of strong time-dependent effects (Appendix S2). Accordingly, its within-subject variance of 41.6±25.7 (mean ± SD) was highest among all subtests (Stack: 26.7±62.5, Turn: 15.0±34.5, Heavy: 7.7±32.3, Light: 3.3±8.4) (Appendix S2). Results of AICc model selection procedures are summarized in Table 2 for each subtest and model. “Pick” and “Stack” tasks had more patients with exponential recovery trajectories (both with and without significant constant; Pick: 79%, Stack: 69%) than the other three tasks. In these two subtests, recovery of most patients (Pick: 59%, Stack: 52%) fell within the category of an exponential curve that converged to normal performance (i.e. within 0±2.5 z-score units, model Exp), whereas linear trajectories were most common in the remaining subtests. Moreover, “Pick” and “Stack” identified patients that did not recover completely (exponential trajectories with a constant below z = −2.5, model ExpC), but this was not the case for “Turn”, “Light” and “Heavy”. In sum, most patients showed a significant recovery with predominantly exponential trajectories in the “Pick” subtest, and 6 did not recover completely in this task. We therefore chose “Pick” as our measure for motor skill recovery.

**Table 2:** Patient classification according to modeled recovery trajectories.

Model	Pick (n)	Stack (n)	Turn (n)	Light (n)	Heavy (n)
ExpC	6	5	0	0	1
Exp	17	15	14	7	2
Lin	6	9	15	22	26

ExpC = exponential model with significant constant outside −2.5 SD (represent incomplete recovery), Exp = exponential model with no significant constant outside −2.5 SD (represents slow complete recovery), Lin = linear model (represents fast complete recovery). n = number of patients.

**Figure 1:**
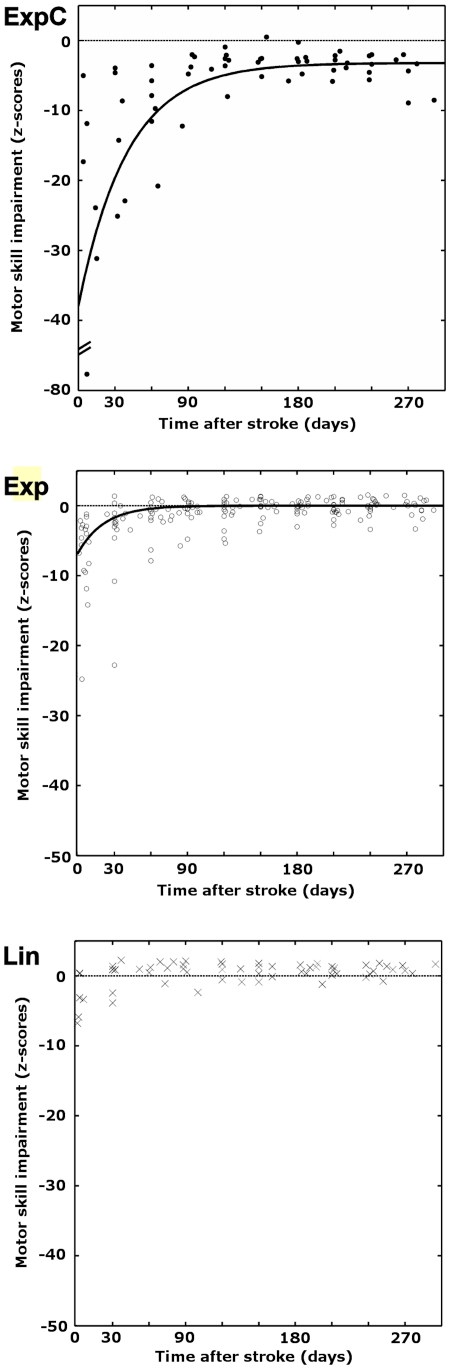
Modeled subgroup recovery trajectories. Scatterplots of subgroup motor impairment scores versus time post-stroke. Impairment scores are in unit standard deviation from the healthy population, where more negative values indicate increasing impairment. Subgroups are named according to the models that best fit their recovery curves. ExpC: patients with slow and impaired recovery (n = 6); Exp: patients with slow and complete recovery (n = 17), and Lin: patients with fast and complete recovery (n = 6). Black solid curves indicate weighted mean recovery trajectories. Dashed line indicates mean of control group performance (z-score = 0).

Table 3 summarizes the modeling results for the “Pick” task. In 23 of 29 patients, individual recovery trajectories were best fit by exponential models, with conditional model evidence *w* ranging from 0.6 to 1.0. In those patients for whom *w* was below 0.9 (n = 9), the next best model was also an exponential one. We chose the model with the highest *w* in these cases. In 6 of these 23 patients, exponential recovery trajectories converged to a z-score below −2.5, indicating incomplete recovery after nine months (subgroup ExpC). The remaining 17 patients showed exponential trajectories that were best fit by models that converged to a z-score 0±2.5, thus being indistinguishable from normal motor performance (subgroup Exp). Finally, 6 patients showed a flat linear trajectory (subgroup Lin). Weighted average parameters (Table 3) of the models in each subgroup were used to compute subgroup recovery trajectories (Fig. 1). Both exponential groups showed moderate to severe initial motor deficits (*I*), whereas the linear subgroup was only mildly affected. Recovery rates *β* did not differ significantly between subgroups ExpC and Exp (U = 48.0, p<.834), but initial deficit *I* did (U = 88.0, p<.008). For subgroup ExpC but not Exp, *I* was significantly correlated with both *β* (one-tailed Spearman's rank correlation, ρ = .829, p<.02) and chronic motor deficit *c* (ρ = .943, p<.002). Since we were interested in the impact of dysfunctional complex somatosensory processing on recovery, we further looked at the correlation between model coefficients and TOR. For group ExpC, there was a significant correlation between *c* and TOR (ρ = .720, p<.001). Of note, patients in subgroup ExpC had highly affected TOR capacities, with a median (and range) of correctly identified objects of 0 (1–4).

**Table 3:** Patient Subgroups Based on Modeled Recovery Trajectories.

Subgroup	Model Parameters										Goodness-of-Fit		
	No.	I	95% CI		β	95% CI		c	95% CI		SSE	AICc	*w*
**ExpC**	4	−10.6	−7.1	−14.1	−0.017	−0.003	−0.031	−2.6	−0.7	−4.6	11.29	17.21	0.75
	6	−31.1	−14.9	−47.3	−0.023	−0.001	−0.045	−3.8	−0.6	−8.3	93.06	36.47	0.97
	13	−51.2	−24.4	−77.9	−0.042	−0.015	−0.069	−3.5	−0.7	−6.2	57.77	35.01	0.90
	14	−16.4	−13.4	−19.4	−0.027	−0.017	−0.038	−2.6	−1.5	−3.7	7.20	12.71	0.97
	17	−93.8	−75.0	−112.5	−0.041	−0.025	−0.058	−6.3	−2.2	−10.3	130.00	41.65	0.83
	18	−10.7	−1.5	−17.8	−0.017	−0.013	−0.047	−3.0	−2.0	−3.9	7.98	11.32	0.93
	**Mean** *	**−34.7**	**−8.8**	**−67.2**	**−0.025**	**−0.017**	**−0.035**	**−3.2**	**−2.3**	**−4.4**			
**Exp**	1	−7.2	−5.6	−8.8	−0.013	−0.004	−0.021				2.61	2.55	0.91
	2	−10.5	−9.0	−12.0	−0.027	−0.021	−0.032				1.17	−7.57	0.97
	3	−2.6	−1.5	−3.7	−0.007	−0.001	−0.012				2.90	−2.38	0.65
	5	−2.8	−1.6	−3.9	−0.047	−0.001	−0.095				0.91	7.99	0.97
	7	−11.1	−8.4	−13.8	−0.061	−0.029	−0.094				2.18	0.76	0.99
	8	−1.7	−1.2	−2.3	−0.018	−0.004	−0.033				0.27	−25.50	0.93
	10	−2.5	−2.0	−3.0	−0.026	−0.015	−0.037				0.16	−25.30	1.00
	12	−5.4	−3.3	−7.4	−0.024	−0.007	−0.040				5.01	3.08	0.69
	19	−5.7	−4.4	−7.1	−0.030	−0.015	−0.045				1.11	1.53	1.00
	20	−7.4	−5.7	−9.1	−0.043	−0.018	−0.069				2.79	3.23	1.00
	21	−6.9	−4.2	−9.7	−0.072	−0.006	−0.138				4.29	1.53	0.57
	22	−4.2	−2.9	−5.6	−0.015	−0.007	−0.022				2.27	−4.84	0.89
	23	−3.6	−2.4	−4.7	−0.059	−0.003	−0.115				1.00	−7.03	1.00
	27	−3.2	−1.5	−4.9	−0.022	−0.004	−0.048				2.00	−0.08	1.00
	26	−14.4	−9.5	−19.2	−0.012	−0.005	−0.019				33.68	22.14	0.74
	28	−13.1	−10.5	−15.5	−0.055	−0.028	−0.071				3.08	−1.78	0.80
	29	−27.6	−19.5	−35.6	−0.020	−0.009	−0.031				98.82	32.91	0.80
	**Mean**	**−6.8**	**−3.7**	**−13.2**	**−0.029**	**−0.020**	**−0.046**						
**Lin**	9	−3.7	−3.2	−4.2	0.016	0.013	0.019				5.23	29.90	0.92
	11	1.0	1.5	0.5	0.003	0.005	0.001				3.85	25.89	0.84
	15	−0.2	1.0	−1.4	−0.001	0.006	−0.008				28.20	41.82	0.80
	16	−0.6	1.0	−2.1	0.007	0.016	−0.002				50.90	50.65	0.69
	24	−2.6	−1.5	−3.7	0.011	0.018	0.004				23.30	40.10	0.98
	25	1.1	1.5	0.7	0.004	0.007	0.001				3.73	24.52	0.47
	**Mean**	**−0.9**	**0.4**	**−2.2**	**0.006**	**0.010**	**0.002**						

Patient subgroups based on whether they showed exponential trajectories converging to an impairment z-score<−2.5 (subgroup ExpC), exponential trajectories converging to an impairment z-score of 0±2.5 (subgroup Exp) or linear trajectories (subgroup Lin). Parameter values are standardized to scores of healthy volunteers; lower values indicate a higher degree of impairment. Model parameters are: I = Intercept (initial deficit), β = exponent (rate of recovery), c = constant (plateau reached at the end of the observation period). Abbreviations: AICc = Akaike Information Criterion with bias correction for small sample sizes; No. = patient number, SSE = model sum of squared errors; w = normalized Akaike weights, i.e. conditional probability of chosen model given the data and the candidate set of models.

*Weighted mean and corresponding 95% CI, i.e. average of individual parameters multiplied with corresponding Akaike weight.

### Lesion Subtraction Maps based on Recovery Subgroups

**Figure 2:**
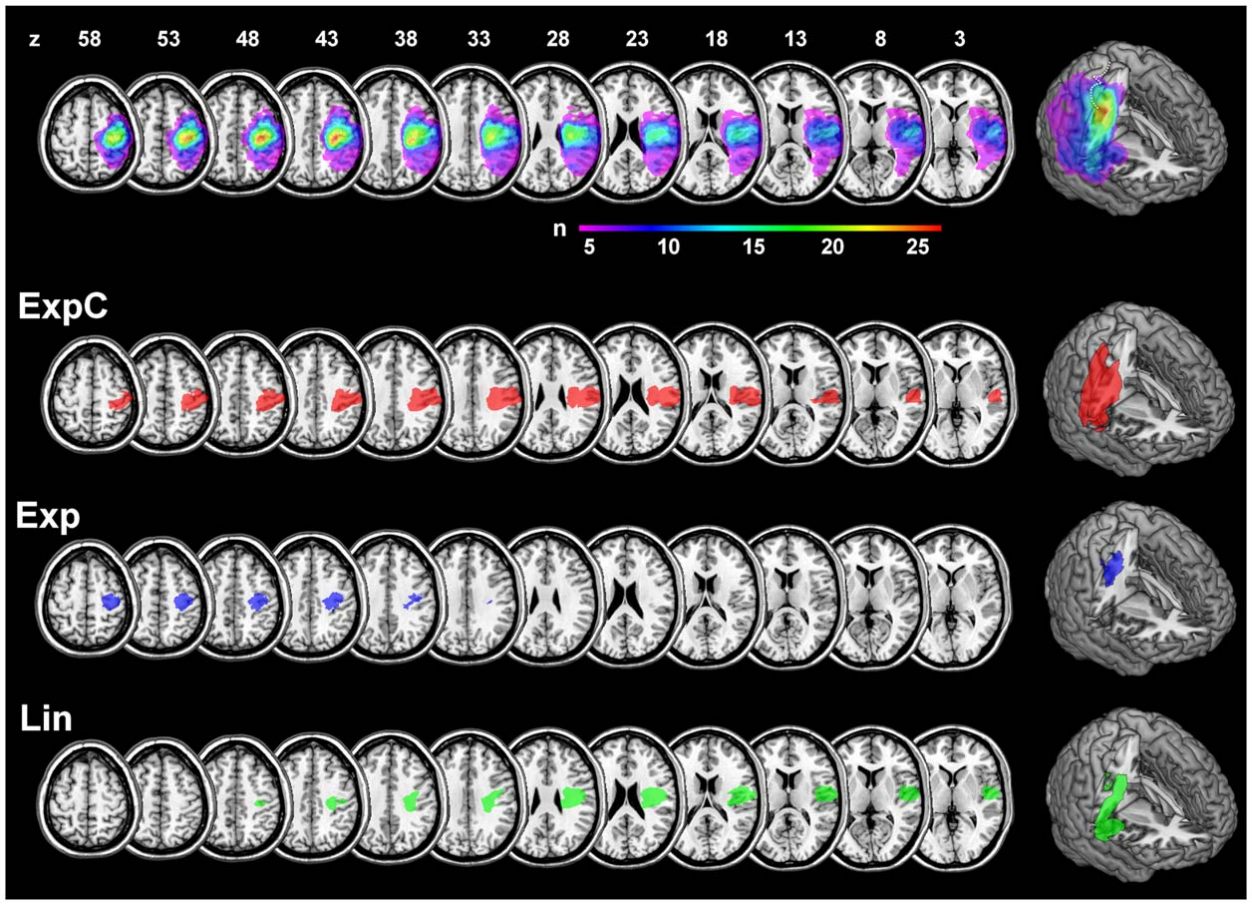
Anatomical overview. Lesion overlap maps for all patients (upper-most row). Rows below show the core maps of subgroups ExpC, Exp and Lin overlaid onto on a standard single-subject brain template. Maps represent voxels were at least 90% of patients overlap (threshold for ExpC and Lin: n = 5; for Exp: n = 15). Images are in neurological convention (left side of the image is left side of the brain) and z-coordinates are given in MNI stereotaxic space. Right-most column shows three-dimensional renderings with a vertical cut through the maximum overlap of the complete cohort.

The lesion map for the complete cohort included 263'360 lesioned voxels (263.4 cm^3^) and showed a maximum lesion overlap in more than 25 patients of 6240 voxels (6.2 cm^3^) in the central sulcus, extending in anterior-inferior direction from the right hand motor area (maximum overlap at MNI: x = 37, y = −23, z = 43) into the underlying subcortical white matter (Fig. 2). For subgroup ExpC, the lesion volume of the core map was 49'317 voxels (49.3 cm^3^), and the center of gravity was located in the depth of the postcentral sulcus (MNI x = 45, y = −3, z = 15). The core map of Exp was smaller (11'269 voxels, 11.3 cm^3^), and its center of gravity lay more anteriorly and superiorly than ExpC in the precentral gyrus (MNI x = 34, y = −2, z = 32). Finally, the center of gravity of the core map for subgroup Lin was shifted inferiorly into the opercular white matter (MNI x = 37, y = −1, z = 8). Across subgroups, lesion volumes were different at a trend level (independent samples Kruskal-Wallis test, H = 6.14, df = 2, p<.047, not significant after removal of one patient with a very large lesion of 266.5 cm^3^ in group ExpC, H = 4.24, df = 2, p<.12).

**Figure 3:**
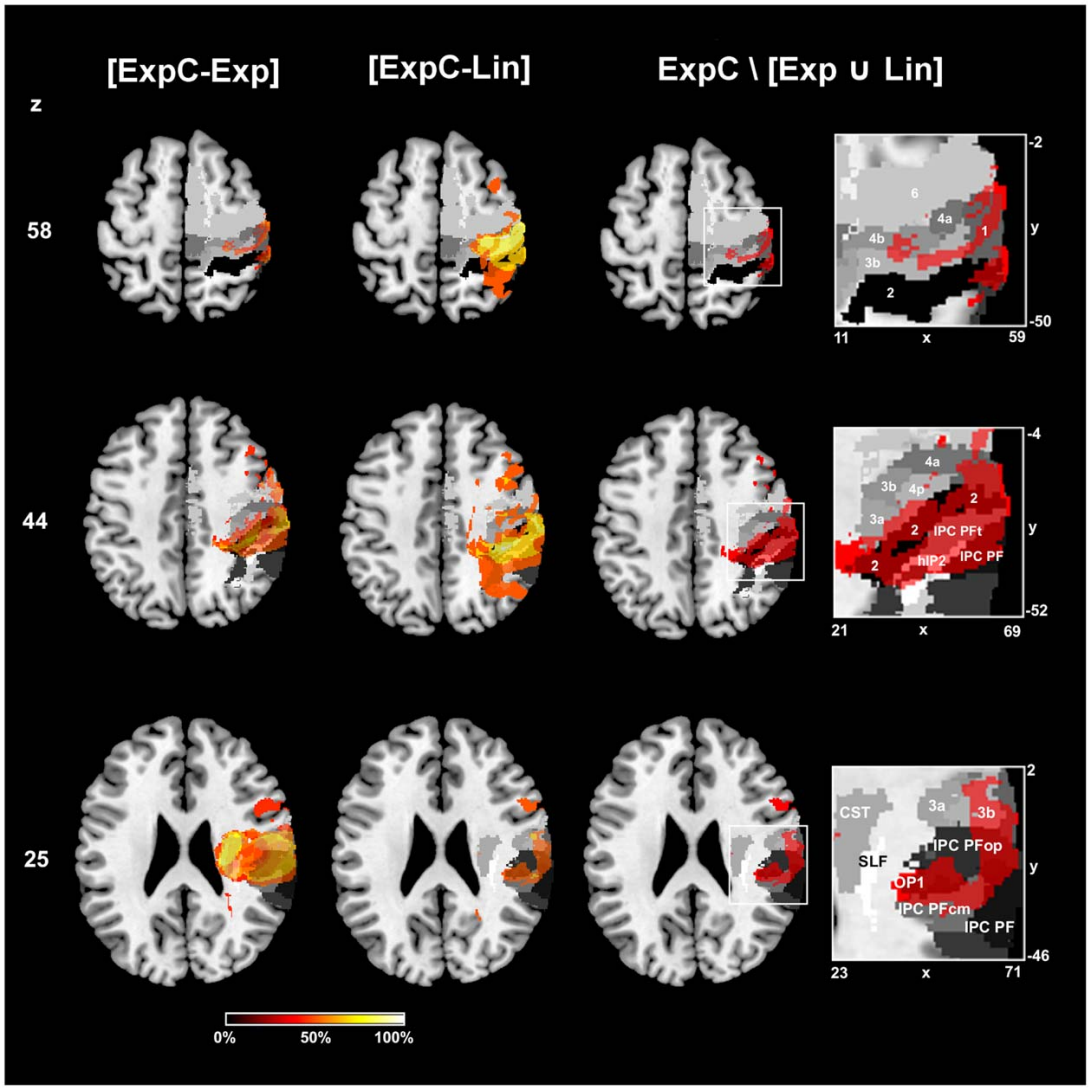
Lesion analysis using subtraction plots and probabilistic cyto- and myeloarchitectonic maps. First and second column from left represent the subtraction of lesion overlap maps from all patients with impaired recovery outcome (ExpC) versus patients with unimpaired recovery (Exp) and complete recovery (Lin), respectively. Voxels lesioned at least 50% more frequently are shown for both subtractions, increasingly brighter colors indicating increased frequency of damage in subgroup A. Third column and magnified views show in monochromatic red the set difference ExpC\[Exp Lin] superimposed onto cyto- and myeloarchitectonic maximum probability maps (i.e. voxels that have the highest probability of belonging to a given area according to the Jülich histological atlas). This set difference corresponds to all voxels that exclusively belong to group ExpC and are thus associated with impaired recovery. Maps are in shades of grey (SLF in white, area 2 in black). All images are in neurological convention and MNI stereotaxic space. Axial slices are at the level of the hand motor area (z = 58), maximum of cortical damage in group ExpC (z = 44) and maximum of subcortical damage in group ExpC (z = 25). Abbreviations: 6 = premotor area 6; 4a/p = primary motor areas; 3a/b, 1, 2 = primary somatosensory areas (anterior to posterior); CST = corticospinal tract; hIP2 = human intraparietal sulcus 2; IPC = Inferior parietal cortex with subareas PF, PFt, PFop, PFcm; OP1 = opercular area 1; SLF = superior longitudinal fascicle. x/y/z = MNI coordinates in mm.

**Mapping of Recovery Outcome:** To identify the regions responsible for impaired recovery outcome, we isolated those voxels that were specifically damaged in subgroup ExpC, but not in the other two groups (since these two recovered completely). We first subtracted the group voxel maps of Exp and Lin from ExpC ([ExpC-Exp] and [ExpC-Lin]). For the delineation of the remainder lesion characterizing group ExpC we then computed the set difference ExpC\[Exp ∪ Lin], which contains all voxels associated with impaired recovery but neither with slow complete (subgroup Exp) nor fast complete recovery (subgroup Lin). Results are shown in Figure 3. The set difference map describing exclusively the lesion of group ExpC, not covered by the lesions of group Exp and group Lin, (thresholded at 50% lesion frequency) comprised 27'214 voxels (27.2 cm^3^) with a centre of gravity in the post-central sulcus, between postcentral gyrus and inferior parietal lobule (MNI: x = 49, y = −27, z = 44) (Fig. 3). The neuroanatomical analysis of this map revealed that it involved the cytoarchitectonic areas of the inferior parietal lobule (affected fraction of area IPC (PFt): 89.9%, area IPC (PFop): 54.9%), the intraparietal sulcus (area hIP1: 65.0%, area hIP2: 63.0%), the primary somatosensory cortex (area 2: 39.4%, area 1: 35.5%, area 3b: 29.6%) and the posterior primary motor cortex (area 4p: 14.5%). These regions were implicated rather centrally (mean ± SD: cr = 1.10±0.07). Of the superior longitudinal fascicle (SLF) 10.1% were damaged centrally (cr = 1.24). Only 0.7% of the CST were affected (cr = 1.14).

**Figure 4:**
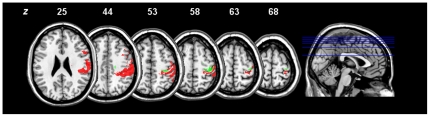
Comparison of lesion maps for impaired recovery outcome and slowed recovery rate. Red areas correspond to voxels that are associated with impaired recovery (i.e. that are lesioned in the patient subgroup ExpC, but not in subgroups Exp and Lin). Green areas encompass those voxels that are damaged in both with exponential recovery subgroups (i.e. that are lesioned in the patient subgroups ExpC and Exp, but not Lin). Images are in neurological convention and MNI stereotaxic space. z = MNI coordinates in mm.

**Mapping of Recovery Dynamics:** To characterize the common lesion to subgroups ExpC and Exp with similar recovery rate but not minimal impairment (subgroup Lin) we computed the intersection [ExpC ∩ Exp] \ Lin. The resultant map contains voxels that are associated with exponential but not linear recovery dynamics. It comprised 1178 voxels (1.18 cm^3^) with a centre of gravity on the pre-central sulcus (MNI: x = 37, y = −25, z = 56), matching the macroanatomical “hand knob” of the single-subject T1 template. Cytoarchitectonic mapping showed that only small proportions of the premotor cortex (area 6: 0.8%), the primary motor cortex (area 4p: 6.6%, area 4a: 2.2%) and the primary somatosensory cortex (area 1: 2.4%, area 3b: 2.1%) were involved, again with central localization (mean centrality ratio for all regions: cr = 1.20±0.23). Results are summarized in Figure 4 (green areas) and compared to the recovery outcome map. Both maps (thresholded at >50% lesion frequency) are clearly separated in anterior-posterior direction along the central sulcus without overlap.

### Lesion extent and centrality across subgroups

Individual lesion extent and centrality are summarized in Table 4. We focus on areas 6, 4a, 4p, 3a, 3b, 1, 2 and hIP1 to 3 because they are known to be involved in skilled hand function, specifically in object manipulation and discrimination (see Discussion). As can be seen from the table, subgroup ExpC was more affected in area 2 and SLF and impaired almost exclusively in all hIP areas (especially hIP1 and hIP2), with rather central location. To rule out that the different recovery pattern seen in subgroup ExpC was due to higher lesion load on the motor system, we compared lesion loads on areas 6, 4a, 4p and CST across all three groups (independent samples Kruskal-Wallis tests, Bonferroni corrected). None of them was significant. Note that, as mentioned above, there was an intersection of the ExpC and Exp groups compared to the Lin group, indicating a higher lesion load in a circumscribed zone involving the areas 4a and 4p with a high cr exactly at the level of the motor hand area.

**Table 4:** Lesion Extent and Localization Relative to Cyto- and Myeloarchitectonic Areas for each Recovery Subgroup.

		Premotor/Primary Sensorimotor Cortex														Intraparietal Sulcus						Tracts			
Subgroup	Vol.§	6		4a		4p		3a		3b		1		2		hIP1		hIP2		hIP3		CST		SLF	
		%	cr	%	cr	%	cr	%	cr	%	cr	%	cr	%	cr	%	cr	%	cr	%	cr	%	cr	%	cr
**ExpC**	73.9 80.7	11.4 17.0	0.8 0.5	12.6 13.3	0.9 0.2	54.3 31.0	1.3 0.2	58.9 21.1	1.1 0.2	62.3 23.2	1.2 0.1	50.3 47.5	1.2 0.3	62.0 40.2	1.2 0.1	86.8 96.6	1.1 0.3	76.7 57.0	1.0 0.4	32.6 72.3	1.0 0.5	33.5 18.9	1.3 0.2	92.9 65.3	1.2 0.4
**Exp**	33.4 116.3	10.0 15.1	1.0 1.4	8.1 7.1	0.9 0.1	31.8 37.6	1.3 0.2	28.0 50.9	1.1 0.5	15.5 35.7	1.2 0.4	7.5 25.1	1.0 0.9	7.6 38.4	1.0 0.9	0.0 0.75	0.0 0.7	0.0 17.7	0.0 1.0	0.0 5.8	0.0 1.1	15.4 28.3	1.2 0.3	6.5 43.8	0.9 1.0
**Lin**	29.3 61.7	1.3 13.4	0.8 1.0	1.6 6.7	0.8 0.5	16.3 24.1	1.3 0.5	21.6 31.7	1.3 0.8	5.6 17.5	1.1 0.9	3.5 4.0	0.6 1.0	1.6 4.6	0.6 1.2	0.0 10.6	0.0 1.3	0.0 0.1	0.0 0.2	0.0 0.3	0.0 0.3	20.5 23.4	1.3 0.3	24.8 47.5	1.1 0.6

Values represent median (upper number in each cell) and interquartile range (lower number in each cell) for percent of area injured by stroke (%) and lesion centrality ratio (cr, where >1 equals central, and <1 peripheral position relative to the affected area. Abbreviations: A, B, C = recovery subgroups, CST = corticospinal tract; hIP = human intraparietal sulcus; SLF = superior longitudinal fasciculus. § = total lesion volume (in cc).

Given our interest in the relationship between motor recovery, damage of somatosensory areas and disruption of somatosensory processing, we correlated performance in TOR with lesion extent on areas 2, hIP2 and the SLF (most damaged in subgroup ExpC) across all patients with exponential recovery. There were highly significant negative correlations between TOR and lesion extent in area 2 (ρ = −.669, p<.001), hIP2 (ρ = −.652, p<.001) and the SLF (ρ = −.451, p<.001), indicating that a higher lesion load in these regions was associated with reduced tactile functions.

### Normalization accuracy

A voxelwise map of the standard deviation showed no asymmetries, indicating no systematic effect of lesions on overall normalization (Appendix S2). The Euclidean distance between template and patient hand motor area was on average 4.9±2.6 mm (mean ± SD), in keeping with previous reports on the precentral anatomical variability and normalization accuracy (Appendix S2). This also confirmed the choice of our smoothing kernel (see Methods above).

## Discussion

The present study yielded three main findings: first, we show that motor skill recovery follows exponential trajectories in a majority of our patients (23 out of 29) with stroke implicating the sensorimotor cortex of the hand area. Second, we present evidence that injury of somatosensory areas within the post-central gyrus and intraparietal sulcus (IPS) decisively affects recovery trajectories, leading to impaired recovery outcome. Accordingly, we found that patients with impaired recovery suffered from severely reduced tactile functions (TOR). Third, patient subgroups with exponential recovery dynamics show a very high lesion load within the motor hand subareas of the precentral gyrus. In summary, recovery outcome and recovery dynamics are reflected by their own distinct lesion map.

Collectively, our results suggest that defective sensorimotor integration has a significant impact on hand motor skills and interferes with the recovery of sensory-guided movements after stroke.

Non-linear patterns of motor recovery after stroke have been reported by several observational studies, both in upper limb and general function [19], [20], [21], [26], but recently also for trunk and lower limb performance [27]. All cited studies indicate that most behavioral recovery occurs within the first 3 months and not much improvement is to be expected beyond 6 months (“recovery plateau”). Of note, this asymptotic pattern was particularly visible in average recovery curves of patient subgroups [20], [21], [26], but not easily identifiable in plots of single-subject trajectories [19]. The recovery plateau seen after 6 months might be attributed to a ceiling effect, since researches often use ordinally scaled test instruments (such as the Action Research Arm Test or the Barthel Index). However, similar recovery patterns have also been reported for tasks that measure motor functions on an interval scale (e.g. grip force, Ref. [26]), that might suffer less from ceiling effects. Thus, “non-linear” or “exponential” patterns of improvement seem to be a consistent feature of post-stroke recovery, and not an artifact of measurement. The mechanisms behind this phenomenon are, to the best of our knowledge, still unidentified. Presumably, the fast early recovery phases are related to processes like reduction of edema, reconstitution of the ischemic penumbra, and resolution of diaschisis, whereas later phases are supported by neuroplasticity and learning mechanisms [9]. However, the exact timing and interaction of these processes in the human brain remain largely unknown.

In our patient cohort, we can confirm the prevalence of non-linear recovery patterns. Interestingly, when looking at each JTT subtest separately, we found that there were striking differences in the magnitude and pattern of recovery between tasks that relied more on distal movements compared to those that relied more on proximal movements. The “Pick” and “Stack” tasks both showed a high proportion of exponential time-courses, as well as a high within-subject variance and a strong time-dependent effect (according to our “3-SD”-criterion). Both require precision grips with thumb, index and middle finger, a configuration that is essential for tactile object exploration [28]. In contrast, “Turn” and the lifting tasks “Light”/“Heavy” had a higher proportion of liner recovery trajectories, less within-subject variance and change over time. This parallels the clinical observation that distal movements usually take longer to recover [9]. Also, recent studies with kinematic recordings show that although speed of grasping (distal) and reaching (proximal) movements recovers similarly over a 90-day period, efficiency in grasping movements (defined as a movement direct to the target) does not recover [29]. This might be due to a loss of selective finger muscle activation and consequently an impairment in finger individuation, as shown by a recent study using surface electromyography [30]. We therefore think that the “Pick” task used as a dependent variable in the present study indeed represents a measure of motor skill dysfunction that specifically reflects loss and recovery of distal motor control, i.e. skilled hand function.

We found that exponential models best described a majority of motor skill recovery trajectories, i.e. a rapid improvement during the first three months that gradually tapered off into a steady-state with (subgroup Exp) or significantly below (subgroup ExpC) normal motor performance. As mentioned, exponential recovery trajectories have been suggested by a number of clinical investigations only at the group-level [19], [20], [21]. Thus, our observation that motor recovery can indeed be modeled with exponential functions at the *individual level* is a novel finding.

The different recovery models allowed us to draw further distinctions. For instance, although subgroups ExpC and Exp differed in chronic impairment, expressed by the constant c in the model of subgroup ExpC, recovery rates (β) did not differ. This indicates that although both groups may share common mechanisms driving recovery dynamics, recovery in subgroup ExpC encounters a limit to further functional gains. Indeed, comparing the lesion maps of all patients with exponential versus those with linear trajectories, we found that exponential recovery dynamics was associated with a circumscribed region that has a high lesion centrality on areas of the primary motor cortex (areas 4a and 4p). Primary motor cortical areas are well-known to be involved in learning novel motor skills [31], [32]. Moreover, a wide range of adaptive plastic changes that support behavioral gains are known to take place within the ipsilesional motor cortex after stroke [31], [33], [34], [35], thus the integrity of this areas and their corticospinal output is clearly important for functional recovery [1], [2], [36]. Lesions centered on the motor cortex plausibly disrupt the effect of such mechanisms. On the other hand, lesion mapping indentified a different set of affected areas associated with impaired recovery outcome. Patients with the persistent motor deficits presented lesions involving areas 2, hIP1/hIP2 and SLF and concomitant severe tactile dysfunction. The individual topographical analysis of these lesions (see Table 4) indicates that this effect was due to a higher lesion load of subgroup ExpC in these areas, both in terms of a larger extent and a higher centrality in comparison to the other two groups. This was associated with a profound tactile dysfunction in this group.

Somatosensory input is essential to accurate hand motor control and skill acquisition [37]. Indeed, disruption of the primary somatosensory cortex by transcranial magnetic stimulation interferes with motor learning [38]. Moreover, motor learning has been shown to be disturbed in chronic stroke patients with proprioceptive deficits [39]. Since stroke recovery probably mobilizes motor learning mechanisms [9], it is conceivable that lesions to somatosensory areas and consecutive somatosensory dysfunction impair such adaptive mechanisms and hinder successful motor skill recovery. Several lines of research indicate that area 2, hIP1 and hIP2, most damaged in our patients with impaired recovery, are part of fronto-parietal networks that are critical for skilled manual behavior [40], [41]. Primate area 2 is densely connected with the primary motor cortex and integrates fine-grained proprioceptive and cutaneous inputs [41], [42], indicating that it provides motor areas with organized information about object shape and texture [43]. Pharmacological inactivation of area 2 leads to severely disorganized grasping movements of the contralesional hand [44], suggesting that it is important for dexterity. Further primate studies have found that the anterior intraparietal sulcus (AIP), which is anatomically connected to the ventral premotor cortex, contains neuron populations which are highly responsive during visual fixation, grasping and manipulation of three-dimensional (3D) objects [45]. These observations are relevant since possible homologues of both area 2 and AIP have been found in human neuroimaging studies. For instance, human area 2 is activated by stimuli such as curvature, edge length and roughness during simple scanning finger movements that do not depend on the object explored (intransitive movements) [46]. In contrast, putative homologues of primate AIP around human IPS show more complex responses, e. g. during visuo-tactile matching tasks [47], somatosensory discrimination [48], [49] and, together with premotor cortices, during skilled manipulation of 3D objects [50]. This kind of task is typically performed with highly coordinated finger movements that are tightly adapted to the object explored (transitive movements). Lesion studies in stroke patients support these regional differences in tactile processing and finger movements: patients with circumscribed lesions in area 2 present severe deficits in discriminating object texture, whereas those with damage to hIP1/hIP2 evidence impaired recognition of three-dimensional shape [6]. Patients with lesion in the parietal lobe show disrupted exploratory finger movements, a gradual decrease in frequency and regularity together with a gradual increase in exploration space depending from the lesion location in its anterior or posterior portion [29]. Taken together, primate and human data show that seamless integration of sensory and motor processing within fronto-parietal networks is crucial for skilled manual behavior. Given that impairment of these areas in our cohort also correlates with TOR, a task that necessitates the integration of multiple sensorimotor processes [16], a disruption of the fronto-parietal network mediating skillful, object-related manual behavior might be at the core of impaired recovery. Further support for this hypothesis is provided by the observed high degree of associated damage to the SLF, which connects frontal and parietal areas [30].

Our findings are in line with a previous longitudinal study by Binkofski et al. [51]. Of note, these authors also indentified three recovery subgroups: the subgroup with the mildest initial deficits invariably had small lesion volumes and most successful outcome, whereas subgroups with moderate or severe initial deficits showed larger lesion volumes and more heterogenous progress (no detail on lesion location was provided). Our results suggest, that lesion location might explain the heterogeneity in these two subgroups. For instance, our subgroup ExpC had also the maximal initial deficit suggesting a critical effect of sensory de-afferentiation even in the acute phase of stroke.

Three apparent criticisms of our analysis might be raised. The first is a problem inherent in using probabilistic maps is that neighboring anatomical areas overlap with low but non-negligible probability. Additional anatomical imprecision might stem from the transformation of the lesion maps into standard anatomic space. We have implemented several steps to reduce potential coregistration errors, e.g. using a robust spatial normalization procedure, smoothing the lesion masks to account for individual anatomical variability and reporting quantitative measures of lesion location appropriate to probabilistic maps. Therefore, we are confident that our anatomical observations concerning individual impairment are robust also with respect to the quality control of the normalization procedure we performed. Another limitation of the probabilisitc atlas is that a few cyto-and myeloarchitectonic maps are still missing from the digital version, notably any thalamo-cortical projections. We can thus not rule out that the latter also had an influence on motor recovery.

The second applies to the analysis of behavioral tasks using exponential time courses, since a ceiling effect has frequently been proposed to explain their occurrence in discrete, descriptive disability scales used in previous studies. We suggest that the choice of the temporal performance of a specific hand function- a continuous, quantitative observable - is not subject to this effect. Thus, the apparent plateau after six months in the temporal performance of the hand function explaining the highest percentage of within-subject variance in longitudinal total JTT scores of our study populations reflects a real bound to clinical recovery. More sensitive laboratory methods, e.g. kinematic recordings of finger movements, might help to discover more subtle impairments in chronic stroke patients [52], [53], [54] but their clinical significance is uncertain.

Finally, as we have focused on a selected population of cortical stroke patients and a restricted set of neuroanatomical areas, our findings represent only one of many aspects that influence recovery.

### Conclusions

To conclude, we found that acute lesions in higher-order somatosensory nodes of fronto-parietal networks engaged in skilled manual behavior impair tactile functions and long-term recovery from hand paresis after stroke. The clinical impact of our results is that patients with impaired tactile abilities represent a population that is unlikely to recover completely and may require long lasting and focused rehabilitation efforts. In contrast, there is some evidence that motor areas are implicated in factors determining the dynamics of the recovery process.

## Supporting Information

Appendix S1 **Appendix S1 contains supplementary methods.** These include a list of cytoarchitectonic areas, details on behavioral testing procedures, recovery modeling, and magnetic resonance image acquisition parameters.
(DOCX)

Appendix S2 **Appendix S2 contains supplementary results concerning behavioral task selection and control of normalization accuracy.**
(DOCX)
