# Supplementary material for: Lesions to Primary Sensory and Posterior Parietal Cortices Impair Recovery from Hand Paresis after Stroke
Source: PLoS One. 2012 Feb 20;7(2):e31275. doi: 10.1371/journal.pone.0031275 (PMC3282712; doi:10.1371/journal.pone.0031275)
Supplement: Appendix S1 — contains supplementary methods. These include a list of cytoarchitectonic areas, details on behavioral testing procedures, recovery modeling, and magnetic resonance image acquisition parameters. (DOCX) [file pone.0031275.s001.docx]

**Appendix S1. Supplementary Methods**

**Cytoarchitectonic Areas**

| Abbreviation | Anatomical region | Reference |
| --- | --- | --- |
| Area 6 | Premotor cortex | Geyer,S. (2003). The Microstructural Border Between the Motor and the Cognitive Domain in the Human Cerebral Cortex (Wien: Springer). |
| Area 4a | Anterior primary motor cortex | Geyer,S. et al., (1996). Nature 382, 805-807 |
| Area 4p | Posterior primary motor cortex | Geyer,S. et al., (1996). Nature 382, 805-807 |
| Areas 3a, 3b, 1 | Primary somatosensory cortex | Geyer,S. et al., (1999). Neuroimage. 10, 63-83. Geyer,S. et al., (2000). Neuroimage. 11, 684-696 |
| Area 2 | Primary somatosensory cortex | Grefkes,C. et al., (2001). Neuroimage. 14, 617-631 |
| Areas hIP1, hIP2, hIP3 | Intraparietal Sulcus | Choi et al. (2006), J. Comp. Neurol. 495, 53-69 Scheperjans et al. (2008a), Cereb. Cortex 18: 846-867 Scheperjans et al. (2008b), Cereb. Cortex 18: 2141-2157 |
| Areas PF(t), PF(op) | Inferior Parietal cortex | Caspers, S et al. (2006), NeuroImage 33, 430-448 Caspers, S et al. (2008), Brain Struct. Funct. 212, 481-495 |

**A. Behavioral Testing Procedures**

**Motor Functions**

*Grip Force*

A Jamar dynamometer was used to measure maximal grip force (GF) over 3 trials, alternating between hands [1] The dynamometer was held in front of the subject with the elbow flexed at 90° and slightly abducted to avoid contact with the trunk. Subjects were instructed to squeeze the lever of the apparatus as strongly as possible without extending the arm. An investigator supported the dynamometer such that the participant could comfortably exert a power grip without the need to additionally stabilize the manipulandum. GF values were recorded in kilograms (higher values indicate better performance).

*Motor skill*

Skilled hand function was measured at both hands using the modified Jebsen-Taylor Test (mJTT), a standardized quantitative assessment of hand function that consists of five timed subtests intended to simulate everyday actions [2]The test is scored by adding the seconds needed to complete each subtest; standard performance norms are available from the original publication for both sexes and for different age groups [2].Several studies have used this test to assess hand function in stroke [3,4,5,6]

The subtests of the mJTT were applied according to the original instructions, as follows: (1) Turning Cards. The subject turned 5 index cards (7.5 cm x 12.5 cm) that were positioned at 12.5 cm from the front end of the table and spaced 5 cm apart, beginning with card farthest away on the opposite side of the used hand. After these two tests, one per hand, a custom-made wooden board (100 x 30 x 3 cm) was placed on the desk at 12.5 cm from the subject for reference. (2) Picking Small Objects. Two paper clips, two bottle caps and two coins were positioned on the same side as the tested hand, touching the board. The subject had to pick each object, starting with the one farthest away and put them into a can positioned at the body midline. (3) Stacking checkers. Four wooden checkers placed against the front of the board were stacked on top of the board. No order was specified. (4) Lifting Light Cans. Five empty aluminum cans (~14 x 9 cm) were placed in front of the board 5 cm apart with the open end facing down, the middle can in front of the subject’s midline. Cans were lifted by the subject and placed on the board, starting with the can farthest away on the same side as the tested hand. (5) Lifting Heavy Cans. The same as subtest 6, but with filled cans (450 g each). mJTT values were recorded in seconds (higher values indicate worse performance).

*Tactile Object Recognition*

Standardized procedure according to Bohlhalter et al. [7]

**B. Modelling Procedures**

**Model Fitting**

Model fits were calculated automatically using the algorithms implemented in the Curve Fitting Toolbox (Version 2.0) for MATLAB 7.8.0 (MathWorks, Natick, Massachusetts). Starting values for intercept and constant were derived by simple observation of the scatter plots of each subject’s scores against time.

**Model Selection**

In the current study, we used linear and exponential models to fit the individual time courses of a motor skill recovery (“recovery trajectory”). We then used model selection procedures based on Akaike’s Information Criterion (AIC;[8,9] ) to select the model that gave the most accurate description of each recovery trajectory. Briefly, AIC reflects the amount of information that is lost when we use any model *m*_i_ to approximate the (biological) process *p* that generated the observed data [10]The AIC for any *m_i_* is given by the equation:

$\mathrm{AIC}_{i}=-2\mathcal{L}_{i}+2k_{i}$ (1)

Here, $\mathcal{L}$ represents the maximum log-likelihood of the model (given the data) and *k* the number of model parameters. If there is a set of different competing models (“candidate set”) that are fit to a data set, the model with minimal information loss, i.e. smallest AIC value, is preferred. AIC model selection seeks to strike a balance between the likelihood of a model and its complexity, given the data and the candidate set of models

AIC can also be calculated from the residual sum of squares (RSS), the number of observations (*n*) and the number of model parameters (*k*) as:

$AIC=ln\left( \frac{\mathrm{RSS}}{n} \right)+2k$ (2)

When the ratio of *n/k* is small (empirically defined as <40), the use of a small sample correction of AIC is recommended (AICc;[11]), as defined by Burnham and Anderson ([8], p. 66):

$\text{AICc}\text{ = AIC +}\frac{\text{2k(k+1)}}{\text{n}\text{-}\text{k}\text{-}\text{1}}$ (3)

Since *n/k* was clearly below 40 in our study (10/4 = 2.5 for the full exponential model) we used Eq. (3), with RSS derived from the curve fitting procedures (see above) for our calculations.

Once we obtained AICc for each model, we ranked them using AICc differences (Δ_i_), which are calculated for *m_i_* as

$\Delta_{i}= \mathrm{AICc}_{i}-\min\mathrm{AIC}c$ (4)

where min AICc is the smallest value in the candidate set, i.e. the AICc value of the best approximating model. Based on these differences, Akaike (as cited in[12]) suggested that exp(-1/2Δ_i_) can be used to obtain and estimate of the relative likelihood for each of the candidate models, given the data. This quantity is used to calculate Akaike weights (*w_i_*) that represent the evidence favoring one model given the data set and the candidate set of models *m_i_*, i=1...R [10,12]

$w_{i}=\frac{exp(-1/2exp\Delta i)}{\sum_{i=1}^{R} exp(-1/2 \Delta r)}$ (5)

A *w_i_* value > 0.9 represents substantial evidence in favor of one model ([8]). Once we had selected the best model or recovery trajectory (in AIC terms), we used *w_i_* to weight each estimated model parameter before averaging them across a subgroup, thus adding model selection uncertainty into our averaged models

Note that selecting models using the AIC paradigm is not a “test” in the traditional sense of hypothesis testing. Therefore, there are no p-values associated with any AIC-related measure [8]Also, note that AIC finds the best fitting model *within the candidate set*, and does not preclude the possibility that there exists another model outside the set that would fit the data even better.

**C. Image acquisition parameters**

**1.5. T MR Images**

The MR measurements were performed on a 1.5-T clinical whole-body MR scanner (Siemens Magnetom Vision) using the standard head coil. The acute study included a conventional fluid attenuated inversion recovery (FLAIR) and T1-weighted axial sequence (T1), an axial diffusion-weighted single-shot echo planar sequence (DWI). T1-images were acquired as follows: T1: TR, 650ms, TE, 14ms, matrix, 512x192, FOV 250x250mm. Parameters for FLAIR were as follows: TR, 8000ms TE, 10 ms,TI, 2000 ms, matrix size, 238 x 256mm;. DWI parameters were: 20 axial slices, slice thickness 5 mm, interslice gap 1.5 mm, TE 103 ms, FOV 240 mm, and matrix 96x128, acquired with b values of 0 and 1000 s/mm2; the high b value DWI measurements were performed with a set of 12 diffusion gradients in the 3 orthogonal (x, y, z) directions in space.

**3.0 T MR Images**

Images were acquired on a 3-T clinical whole-body MR scanner (Simens Trio). The parameters for the T1 images were: TR, 400 ms; TE, 12 ms; matrix size, 144 x 256; FOV, 177 x 230 mm, for the fluid-attenuated inversion recovery (FLAIR) images TR, 7620 ms; TE, 82 ms; matrix, 156x256; FOV, 177x230 mm, and for the DWI studies TR, 3100 ms; TE, 106 ms; matrix, 128x128; FOV, 196x196 mm; and 2 b-values (1000 and 2000 s/mm2) using a set of 12 gradients. The latter applied in each of the 3 principal gradient directions (x, y, and z) were used to calculate the apparent diffusion coefficient (ADC).

**REFERENCES**

1. Mathiowetz V, Kashman N, Volland G, Weber K, Dowe M, et al. (1985) Grip and pinch strength: normative data for adults. Arch Phys Med Rehabil 66: 69-74.

2. Jebsen RH, Taylor N, Trieschmann RB, Trotter MJ, Howard LA (1969) An objective and standardized test of hand function. Arch Phys Med Rehabil 50: 311-319.

3. Chestnut C, Haaland KY (2008) Functional significance of ipsilesional motor deficits after unilateral stroke. Arch Phys Med Rehabil 89: 62-68.

4. Sunderland A (2000) Recovery of ipsilateral dexterity after stroke. Stroke 31: 430-433.

5. Sunderland A, Bowers MP, Sluman SM, Wilcock DJ, Ardron ME (1999) Impaired dexterity of the ipsilateral hand after stroke and the relationship to cognitive deficit. Stroke 30: 949-955.

6. Wetter S, Poole JL, Haaland KY (2005) Functional implications of ipsilesional motor deficits after unilateral stroke. Arch Phys Med Rehabil 86: 776-781.

7. Bohlhalter S, Fretz C, Weder B (2002) Hierarchical versus parallel processing in tactile object recognition: a behavioural-neuroanatomical study of aperceptive tactile agnosia. Brain 125: 2537-2548.

8. Burnham K AD (2010) Model Selection and Multi-Model Inference: A practical information-theoretic approach. New York: Springer.

9. Akaike H (1974) A new look at the statistical model identification. IEEE Trans Aut Control 19: 7.

10. Posada D, Buckley TR (2004) Model selection and model averaging in phylogenetics: advantages of akaike information criterion and bayesian approaches over likelihood ratio tests. Syst Biol 53: 793-808.

11. Hurvich CM, Tsai CL (1995) Model selection for extended quasi-likelihood models in small samples. Biometrics 51: 1077-1084.

12. Wagenmakers EJ, Farrell S (2004) AIC model selection using Akaike weights. Psychon Bull Rev 11: 192-196.
