# Supplementary material for: Lesions to Primary Sensory and Posterior Parietal Cortices Impair Recovery from Hand Paresis after Stroke
Source: PLoS One. 2012 Feb 20;7(2):e31275. doi: 10.1371/journal.pone.0031275 (PMC3282712; doi:10.1371/journal.pone.0031275)
Supplement: Appendix S2 — contains supplementary results concerning behavioral task selection and control of normalization accuracy. (DOCX) [file pone.0031275.s002.docx]

**Appendix S2. SUPPLEMENTARY RESULTS**

**Task selection**

Figure S1 summarizes the longitudinal cumulative (CumVar) and task variance between stroke patients. As can be seen from the chart, the task Picking Small Objects (PSO, Pick), had the largest variance of all subtests across the observation period.

**Figure S1: Longitudinal between-subjects variance**

Cumulative variance (CumVar) for each task at each month (M0-9) post stroke. As can be seen from the chart, the task Picking Small Objects (Pick), had the largest variance of all subtests across the observation period.

Table S1 and S2 below summarize the average variance of each task both between-subjects (i.e. variance per visit across patients for each task, as in Fig. S1) and within-subjects (i.e. per patient across visits for each task), respectively. The Picking Small Objects task varies greatly in both dimensions.

**Table S1: Average between-subjects variance**

| *Task* | *%CumVar* | *SD* |
| --- | --- | --- |
| Pick | 42.38 | 13.88 |
| Stack | 21.61 | 4.48 |
| Turn | 16.55 | 4.59 |
| Heavy | 10.28 | 5.84 |
| Light | 9.18 | 4.21 |

Percent of cumulative variance (%CumVar) and its standard deviation (SD) per visit across patients for each task

**Table S2: Average within-subjects variance**

| *Task* | *Var* | *SD* |
| --- | --- | --- |
| Pick | 41.64 | 98.66 |
| Stack | 26.56 | 62.85 |
| Turn | 14.95 | 35.24 |
| Light | 7.66 | 32.27 |
| Heavy | 3.35 | 8.37 |

Percent of cumulative variance (%CumVar) and its standard deviation (SD) per patient across visits for each task

**Normalization accuracy**

We first calculated the voxel-wise standard deviation between the single subject T1-template of SPM8 with each patient’s T1-image using the image calculator (ImCalc) in SPM8. Results are summarized in Figure S2. Furthermore, we calculated the Euclidean distance between the visually indentified hand knob from the single subject T1-template of SPM8 and the center of the visually indentified hand knob from each single patients T1 scan. The Euclidean distance was defined as in space as

$$d\left( p,t \right)=\sqrt{\left( px-tx \right)\text{2}+\left( py-ty \right)\text{2}-\left( pz-tz \right)\text{2}}$$

Were d denotes distance between the patient (p) and template (t) coordinates (x,y,z) in MNI space. The hand knob of the template was arbitrarily defined as MNI x=-30.80, y =-26.80, z= 55.20. To search for the hand knob in each patient, the axial slice was chosen at z =55.20 and the hand knob was identified starting from this slice and moving slice by sliche first in cranio-caudal, then in anterior-posterior and finally in medial-lateral direction. The hand knob could be unequivocally identified in 27 /29 patients. One had an epsilon-shaped hand knob (a common variant), one had none. Results are summarized in table S3.

**Figure S2 Voxel-wise standard deviation map**

**
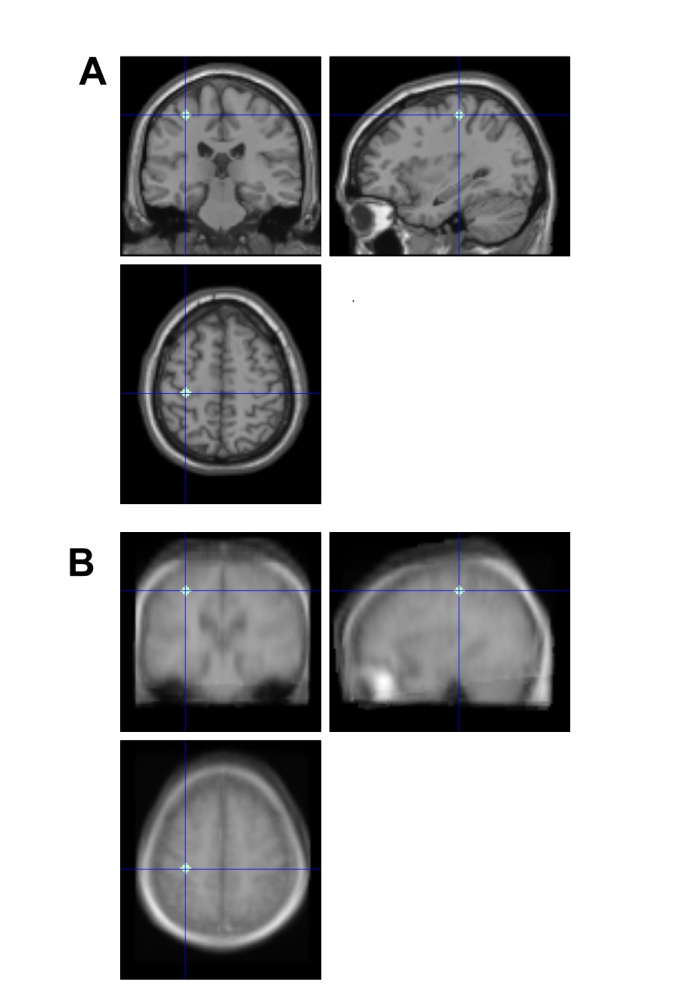
**

**
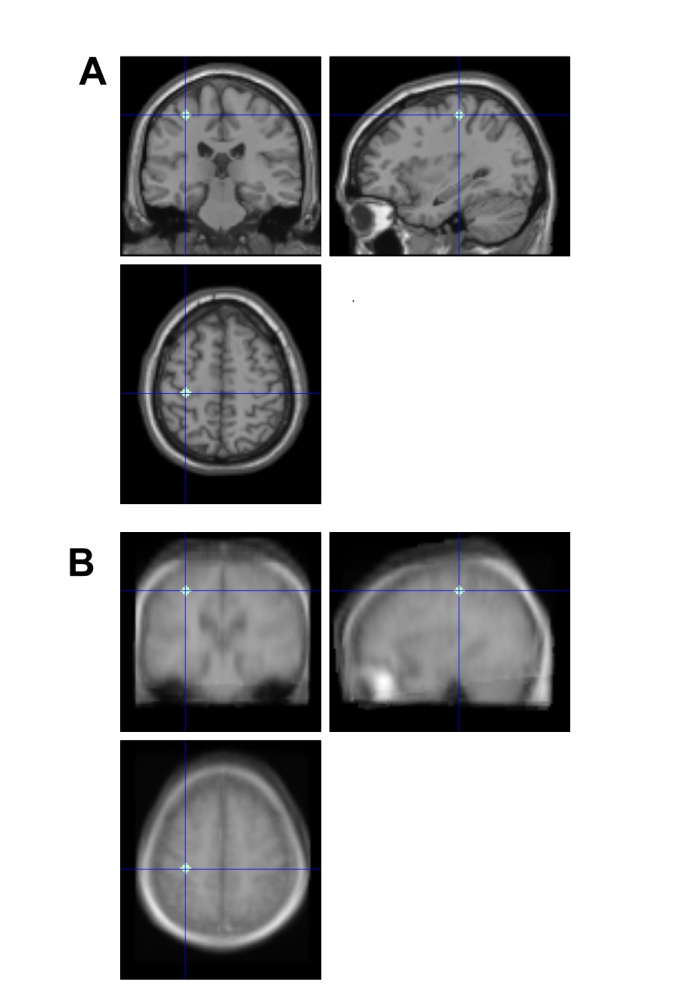
**

**Panel A.** Single subject T1 template from SPM8 **Panel B** Voxel-wise standard deviation map. Images are in neurological convention in coronal, sagittal and axial projection (clockwise). Images are neurological convention (left on the readers side equals left side of the brain). Crosshairs are at MNI coordinates x=-30.80, y =-26.80, z= 55.20

**Table S3 Euclidean distance between patient and template hand knob.**

|  | px | py | pz | tx | ty | tz | d |
| --- | --- | --- | --- | --- | --- | --- | --- |
| p01 | -28.20 | -22.80 | 56.00 | -30.80 | -26.80 | 55.20 | 4.84 |
| p02 | -30.80 | -20.40 | 55.20 | -30.80 | -26.80 | 55.20 | 6.40 |
| p03 | -32.40 | -22.80 | 55.10 | -30.80 | -26.80 | 55.20 | 4.31 |
| p04 | -34.80 | -24.40 | 56.20 | -30.80 | -26.80 | 55.20 | 4.77 |
| p05 | -31.60 | -19.60 | 55.40 | -30.80 | -26.80 | 55.20 | 7.25 |
| p06 | -30.00 | -26.00 | 55.30 | -30.80 | -26.80 | 55.20 | 1.14 |
| p07 | -34.80 | -23.60 | 55.20 | -30.80 | -26.80 | 55.20 | 5.12 |
| p08 | -31.60 | -24.40 | 57.60 | -30.80 | -26.80 | 55.20 | 3.49 |
| p09 | -30.80 | -24.40 | 54.40 | -30.80 | -26.80 | 55.20 | 2.53 |
| p10 | -32.40 | -22.80 | 55.30 | -30.80 | -26.80 | 55.20 | 4.31 |
| p11 | -31.60 | -19.60 | 53.60 | -30.80 | -26.80 | 55.20 | 7.42 |
| p12 | -32.40 | -19.60 | 55.20 | -30.80 | -26.80 | 55.20 | 7.38 |
| p14 | -30.80 | -25.80 | 54.20 | -30.80 | -26.80 | 55.20 | 1.41 |
| p15 | -30.00 | -26.80 | 57.60 | -30.80 | -26.80 | 55.20 | 2.53 |
| p16 | -34.80 | -25.20 | 55.20 | -30.80 | -26.80 | 55.20 | 4.31 |
| p17 | -32.40 | -21.20 | 52.00 | -30.80 | -26.80 | 55.20 | 6.65 |
| p19 | -34.80 | -21.20 | 55.20 | -30.80 | -26.80 | 55.20 | 6.88 |
| p20 | -30.80 | -26.90 | 55.20 | -30.80 | -26.80 | 55.20 | 0.10 |
| p24 | -38.00 | -24.40 | 55.30 | -30.80 | -26.80 | 55.20 | 7.59 |
| p25 | -35.60 | -26.80 | 55.20 | -30.80 | -26.80 | 55.20 | 4.80 |
| p26 | -31.60 | -24.40 | 55.20 | -30.80 | -26.80 | 55.20 | 2.53 |
| p27 | -22.80 | -24.40 | 55.20 | -30.80 | -26.80 | 55.20 | 8.35 |
| p31 | -28.40 | -23.60 | 55.10 | -30.80 | -26.80 | 55.20 | 4.00 |
| p33 | -30.80 | -23.60 | 66.10 | -30.80 | -26.80 | 55.20 | 11.36 |
| p35 | -27.60 | -31.60 | 61.60 | -30.80 | -26.80 | 55.20 | 8.62 |
| p36 | -30.80 | -26.80 | 56.80 | -30.80 | -26.80 | 55.20 | 1.60 |
| p37 | -30.80 | -27.80 | 52.80 | -30.80 | -26.80 | 55.20 | 2.60 |
|  |  |  |  |  |  | Mean | 4.90 |
|  |  |  |  |  |  | SD | 2.64 |

Abbreviations: px/y/z = patient coordinates, tx/y/z template coordinates in MNI space (mm). d = euclidean distance (mm).
